# FAMILY CAREGIVERS’ RESPONSES TO DAILY BEHAVIORAL SYMPTOMS OF DEMENTIA: THE MODERATING ROLE OF RELATIONSHIP QUALITY

**DOI:** 10.1093/geroni/igz038.2210

**Published:** 2019-11-08

**Authors:** Richard E Chunga, Yin Liu, Kyungmin Kim, Steven H Zarit

**Affiliations:** 1 University of Massachusetts Boston, Boston, Massachusetts, United States; 2 Utah State University, Logan, Utah, United States; 3 Pennsylvania State University, University Park, Pennsylvania, United States

## Abstract

Providing care for persons with dementia (PWD) is frequently regarded as highly stressful, but how caregivers perceive care-related stressors depends on a variety of contexts. Research has demonstrated that relationship quality between the caregiver and receiver – as an important dyadic context – can influence the magnitude of this perceived distress. Using 8-day diary data from 173 family caregivers of PWD (day N = 1,359), this study examined the moderating effect of relationship quality on caregivers’ stress responses to daily behavioral and psychological symptoms of dementia (BPSD), comparing within- and between-person effects. Multilevel models suggested differences in the association between BPSD occurrence and perceived distress of BPSD (i.e., negative within-person association, but positive between-person association). However, we found that both associations were moderated by relationship quality; that is, better dyadic relationship quality seemed to be protective against distress at both within- and between-person levels.

